# Experimental Study on Tensile Mechanical Properties and Reinforcement Ratio of Steel–Plastic Compound Geogrid-Reinforced Belt

**DOI:** 10.3390/ma14205963

**Published:** 2021-10-11

**Authors:** Qingbiao Wang, Yue Li, Hongxu Song, Jianing Duan, Zhongjing Hu, Fuqiang Wang, Haolin Xu, Zhengyin Liu, Mingjing Zhang, Liming Wang, Yeming Wu, Zhenyue Shi

**Affiliations:** 1 State Key Laboratory of Mining Disaster Prevention and Control Co-Founded by Shandong Province and the Ministry of Science and Technology, Shandong University of Science and Technology, Qingdao 266590, China; skd990748@sdust.edu.cn; 2 National Engineering Laboratory for Coalmine Backfilling Mining, Shandong University of Science and Technology, Tai’an 271019, China; 3 College of Resources, Shandong University of Science and Technology, Tai’an 271019, China; 202083300005@sdust.edu.cn (J.D.); 202083300024@sdust.edu.cn (H.X.); 4 College of Civil Engineering and Architecture, Shandong University of Science and Technology, Qingdao 266590, China; 201983040032@sdust.edu.cn (Y.L.); 201983040061@sdust.edu.cn (F.W.); 5 College of Energy and Mining Engineering, Shandong University of Science and Technology, Qingdao 266590, China; 201983010016@sdust.edu.cn; 6 College of Safety and Environmental Engineering (College of Safety and Emergency Managemen), Shandong University of Science and Technology, Qingdao 266590, China; huyang@sdust.edu.cn; 7 Shandong Provincial Communications Planning and Design Institute Group Co., Ltd., Jinan 250031, China; liuzhengyin009@126.com (Z.L.); sdjtyyt@126.com (M.Z.); 8 Shandong Dageng Project Material Co., Ltd., Tai’an 271600, China; wlm13705482778@126.com (L.W.); wym13563835879@126.com (Y.W.)

**Keywords:** steel–plastic compound geogrid-reinforced belt, tensile test, minimum reinforcement ratio, optimum reinforcement ratio

## Abstract

The steel–plastic compound geogrid has been widely used as a new reinforcement material in geotechnical engineering and other fields. Therefore, it is essential to fully understand the mechanical properties of steel–plastic compound geogrid-reinforced belts to utilize steel–plastic compound geogrids efficiently. In this study, tensile mechanical tests of steel wire, polyethylene geogrid belt, and steel–plastic compound geogrid-reinforced belt were conducted with respect to the tensile mechanical properties of steel–plastic compound geogrid-reinforced belts. In addition, the minimum reinforcement and optimal reinforcement ratios of steel–plastic compound geogrid-reinforced belts were summarized. The results showed that the steel–plastic compound geogrid-reinforced belts possessed an incongruent force of the internal steel wire during the tensile process. The tensile stress–strain curve of the steel–plastic compound geogrid-reinforced belt can be divided into the composite adjustment, steel wire breaking, and residual deformation stages. The tensile strength of the steel–plastic compound geogrid-reinforced belt is proportional to the diameter and number of steel wires in the reinforced belt. The minimum and optimum reinforcement ratios of steel wire in the steel–plastic compound geogrid-reinforced belt were 0.63% and 11.92%, respectively.

## 1. Introduction

**Figure 1:**
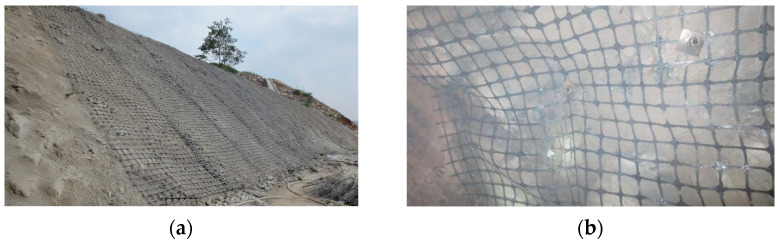
Engineering application of a steel–plastic compound geogrid: (**a**) slope support (**b**) roadway support.

With the development of engineering technology and improvement of material performance, all types of geogrids have gradually occupied an increasingly important position in present engineering construction owing to their excellent material performance advantages [1,2,3,4,5,6,7,8]. The steel–plastic compound geogrid is one of the main representatives, as shown in Figure 1.

**Figure 2:**
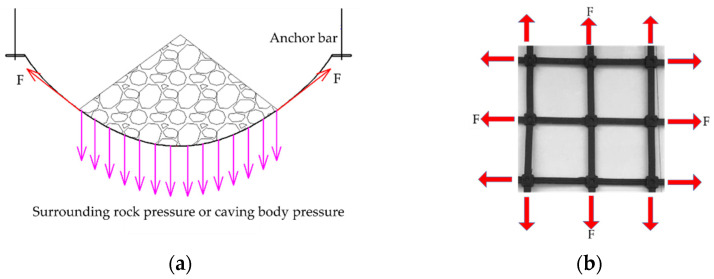
The tensile stress analysis of steel–plastic compound geogrid in an anchor mesh support: (**a**) stress analysis chart of the anchor mesh support; (**b**) tensile stress analysis chart of the steel–plastic compound geogrid.

In anchor mesh support technology and other engineering fields, steel–plastic compound geogrid is a new highly efficient composite reinforcement material owing of its high tensile strength to withstand the load from rock and soil, as shown in Figure 2. Therefore, the tensile strength and its deformation characteristics are important indicators for evaluating the performance of steel–plastic compound geogrids and for guaranteeing the engineering safety.

A number of scholars have conducted studies on the tensile mechanical properties of various geogrids, such as steel–plastic compound geogrids.

Wang et al. [9] analyzed the tensile strength of a steel–plastic compound geogrid mesh surface through laboratory tests and engineering applications and verified the effectiveness of the steel–plastic compound geogrid for engineering support. Peng et al. [10,11] performed tensile tests on different specifications of steel–plastic compound geogrids. The effects of the tensile rate and tensile fixture on the tensile properties of steel–plastic compound geogrids were confirmed by comparing the tensile strength of the geogrids in different experimental groups. Cardile et al. [12] conducted a series of monotonic and multistage tensile tests on high-density polyethylene unidirectional extrusion geogrids and analyzed the influence of cyclic tensile loading history (by changing the pre-stressed tensile load, frequency, amplitude, and number of cycles) on the characteristic parameters of the hysteresis curve (that is, the maximum and residual strains accumulated during each cyclic loading, tensile stiffness, and area of the hysteresis loops). Cho et al. [13] used ASTM 06637 and ISO 10319 test methods to carry out wide-width tensile tests under different sample lengths and tensile rates and studied the wide-width tensile strength performance of a geogrid. Dong et al. [14] used the fast Lagrangian analysis of continua (FLAC) numerical software to study the response of rectangular and triangular aperture geogrids under uniaxial tensile loads in different directions and evaluated the influence of the aperture shape, elastic modulus, and cross-sectional area of geogrids on the tensile stiffness of geogrids. Perkins et al. [15] conducted a series of wide-width uniaxial tensile tests on biaxial geogrids to study the elastic response of geogrids under cyclic and continuous tensile loads. The results demonstrated that there was insignificant difference in the resilient modulus between the different investigated load forms. Han et al. [16] conducted a series of wide-range tensile tests on several geogrids and established the relationship between the tensile strength and strain of various geogrids. Wang et al. [17] studied the mechanical properties of glass-fiber-reinforced plastic geogrid through the loading speed, temperature tensile test, and FLAC 3D numerical simulation and obtained the mechanical parameters of the displacement time curve, fracture strength, and elongation at break. The results indicated that the fracture strength of the geogrid was closely related to the temperature and loading rate. Yoo et al. [18] introduced a wide-width tensile test of a geogrid under different loading rates and studied the effect of the tensile strain rate on the geogrids deformation behavior.

An analysis of the literature identified several studies that have primarily focused on the influence of different experimental conditions on the tensile properties of the geogrid mesh surface. However, only a few studies have been reported on the reinforcement belt, constituting the basic unit of the mesh surface. In the steel–plastic compound geogrid, the reinforced belt is composed of a steel wire and polyethylene material, generating the complexity in the mechanical characteristics of the composite-reinforced belt. In this regard, a failure to fully apprehend the tensile mechanical properties of steel–plastic compound geogrid-reinforced belts directly affects the utilization benefits of steel–plastic compound geogrid and the safety and stability of reinforcement projects. Therefore, it is necessary and important to study the tensile mechanical properties of steel–plastic compound geogrid-reinforced belts.

In this study, the method of combining experiment and theory was employed. Initially, indoor tensile tests were performed on the three materials of cold-drawn non-alloy steel wire for springs, polyethylene geogrid belt, and steel–plastic compound geogrids-reinforced belt, to explore the mechanical properties of the three materials under tensile force. Subsequently, the composite mechanical characteristics of the steel–plastic compound geogrid-reinforced belt were analyzed by measuring the steel wire reinforcement ratio of the steel–plastic compound geogrid-reinforced belt as a variable. Finally, the minimum and optimal reinforcement ratios of steel wire in the steel–plastic compound geogrid-reinforced belt were studied based on the tensile test results. The results of this study have important practical significance for studying the steel–plastic compound geogrid and its engineering reinforcement field.

## 2. Materials and Methods

### 2.1. Testing Materials

The testing required three types of test materials: cold-drawn non-alloy steel wire for springs, polyethylene geogrid belt, and steel–plastic compound geogrid-reinforced belt. The three materials were produced by Shandong Runde Engineering Materials Co., Ltd. (Tai’an, China). The material parameters of cold-drawn non-alloy steel wire for springs and polyethylene geogrid belt are similar to those of the steel–plastic compound geogrid-reinforced belt.

**Figure 3:**
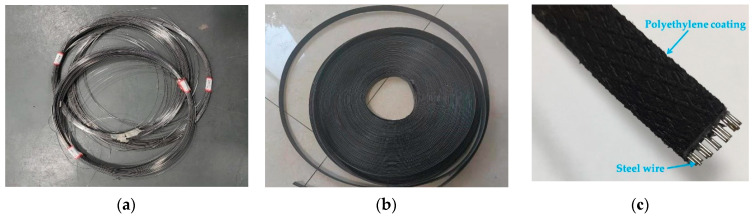
Sample images of the test materials: (**a**) steel wire; (**b**) polyethylene geogrid belt; (**c**) steel–plastic compound geogrid-reinforced belt.

A cold-drawn non-alloy steel wire for springs is shown in Figure 3a. The polyethylene geogrid belt has polyethylene as a raw material with a certain added amount of anti-ultraviolet, anti-aging additives, and other enhanced material extrusion moldings, as shown in Figure 3b. The steel–plastic compound geogrid-reinforced belt has the cold-drawn non-alloy steel wire for springs as the skeleton, polyethylene as the matrix, and a certain amount of anti-ultraviolet, anti-aging additives, and other reinforcing substances, which are extruded and compounded, as shown in Figure 3c.

### 2.2. Test Equipment and Scheme

#### 2.2.1. Test Equipment

A microcomputer-controlled electro-hydraulic servo universal testing machine was used as the tensile test equipment for the steel wire, polyethylene geogrid belt, and steel–plastic compound geogrids-reinforced belt. The YYU-5/50 extensometer produced by the Central Iron & Steel Research Institute was used in the stress and strain test device.

**Figure 4:**
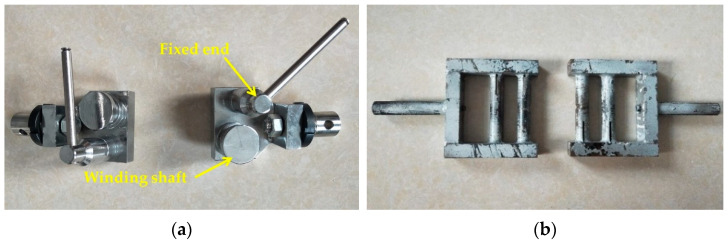
Pictures of the special fixture for the test: (**a**) special fixture for winding the steel wire; (**b**) special fixture for winding the reinforced belt.

Slip or clamping damage of the steel wire easily occurs in the process of the traditional fixture test because the steel wire diameter used in this test is small. Therefore, a special fixture for the winding steel wire was designed in this experiment to solve the above problems, as shown in Figure 4a. Because the steel wire is contained in the steel–plastic compound geogrid-reinforced belt, the clamping force of the ordinary flat fixture is insufficient, which easily causes the end of the reinforced belt to slide out. Moreover, the clamping force of the extrusion fixture is very large, leading to the wire drawing phenomenon of the outer wrapping layer of the reinforced belt due to damage. Therefore, a special fixture for a winding reinforced belt was designed to meet the test requirements. The basic principle of the special fixture for the winding reinforced belt was to overcome the damage of the clamp on the experimental material by winding the reinforced belt, as shown in Figure 4b.

#### 2.2.2. Experimental Group Design

At present, the steel–plastic compound geogrid primarily utilizes three types of cold-drawn non-alloy steel wires for springs of 0.5 mm, 0.6 mm, and 0.7 mm. Therefore, in this study, these three types of steel wires and the steel–plastic compound geogrid using these three steel wires as the skeleton were employed for the comparative experiments in this study.

The strength of the steel–plastic compound geogrid-reinforced belt is directly related to the reinforcement ratio. Therefore, the reinforcement ratio was set as an independent variable in the experiment of steel–plastic compound geogrid-reinforced belt. The reinforcement ratio is calculated as follows:

(1) $$\lambda_{s}=\frac{S_{s}}{S_{c}}=\frac{S_{1}\times n}{b\times h}=\frac{\pi \times {\left(\frac{d}{2}\right)}^{2}\times n}{b\times h}=\frac{\pi \times d^{2}\times n}{4\times b\times h}$$

where *λ_S_* is the reinforcement ratio, that is, the section area of the steel wire in the steel–plastic compound geogrids-reinforced belt, accounting for the proportion of the total section area of the reinforced belt. *S_S_*, *S_c_*, and *S*_1_ are the cross-sectional area of the steel wire in the reinforced belt, total cross-sectional area of the steel–plastic composite geogrids-reinforced belt, and cross-sectional area of a single steel wire, respectively. *n* is the number of steel wire roots in the steel–plastic compound geogrid-reinforced belt. *b* and *h* are the width and thickness of the sectional area of the reinforced belt, respectively. *d* is the diameter of a single steel wire in a reinforced belt.

Six groups of control experiments were conducted for different experimental groups of cold-drawn non-alloy steel wire for springs, polyethylene geogrid belt, and steel–plastic compound geogrids-reinforced belt to study the mechanical properties of the steel–plastic compound geogrid-reinforced belt. The detailed design of the experimental groups is specified in Table 1.

#### 2.2.3. Test Method

By referring to the standard [19,20,21], tensile tests of cold-drawn non-alloy steel wire for springs, polyethylene geogrid belt, and steel–plastic compound geogrid-reinforced belt were performed using the following methods. The samples were placed at 23 ± 5 °C for at least 24 h and tested in this environment. The original standard distance of the test material was set to 20–25 cm, and the tensile loading rate was 10 mm/min. Each test material was pre-tensioned before the formal tensile test, and the pre-tension was 1% of the maximum load. The pre-tension information for the three materials is as follows:

1. According to the standard requirements of “cold-drawn non-alloy steel wire for springs” [20], the stress, *σ_S_*, of cold-drawn non-alloy steel wire for springs is approximately 2000–2400 MPa. Therefore, the pre-tension tension and force can be adjusted according to the diameter of the wire. The maximum force and pre-tension force of the steel wire are finally obtained through calculation, as shown in Table 2.
2. The composition, production process, and shape characteristics of polyethylene products have a significant impact on the tensile strength. Therefore, it is essential to ensure the accuracy and rigor of the test, according to the specifications [21], before the formal tensile test of the polyethylene geogrid belt. Three groups of pre-experiments were conducted to obtain the estimated maximum force of the polyethylene geogrid belt, and the required pre-tension value was obtained, as shown in Table 3.
3. The tensile force of the steel–plastic compound geogrids-reinforced belt is mainly borne by the steel wire inside the reinforced belt. Therefore, the maximum force estimation of the steel–plastic compound geogrids-reinforced belt is the resultant force of all steel wires in the reinforced belt. Subsequently, the pre-tension value of the steel–plastic compound geogrids-reinforced belt was calculated, as shown in Table 4.

**Figure 5:**
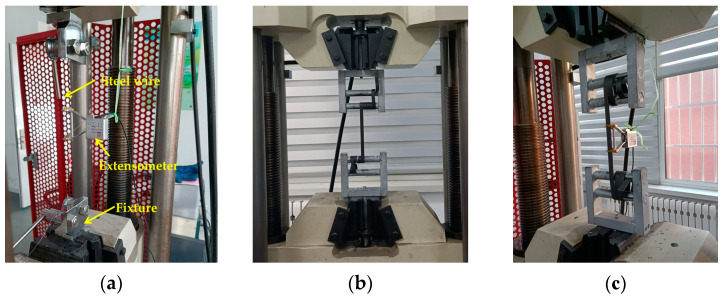
Tensile tests of cold-drawn non-alloy steel wire for springs, polyethylene geogrid belt, and steel–plastic compound geogrids-reinforced belt: (**a**) tensile test of cold-drawn non-alloy steel wire for springs; (**b**) tensile test of polyethylene geogrid belt; and (**c**) tensile test of steel–plastic compound geogrids-reinforced belt.

The specimen was cleared after it reached pre-tension, and the length of the specimen was measured. Subsequently, the formal tensile test was initiated, and the stress, strain, and tensile force of the cold-drawn non-alloy steel wire for springs, polyethylene geogrid belt, and steel–plastic compound geogrid-reinforced belt were recorded. The tensile test process of cold-drawn non-alloy steel wire for springs, polyethylene geogrid belt, and steel–plastic compound geogrid-reinforced belt is shown in Figure 5.

## 3. Results

### 3.1. Tensile Test Results of Cold-Drawn Non-Alloy Steel Wire for Springs

**Figure 6:**
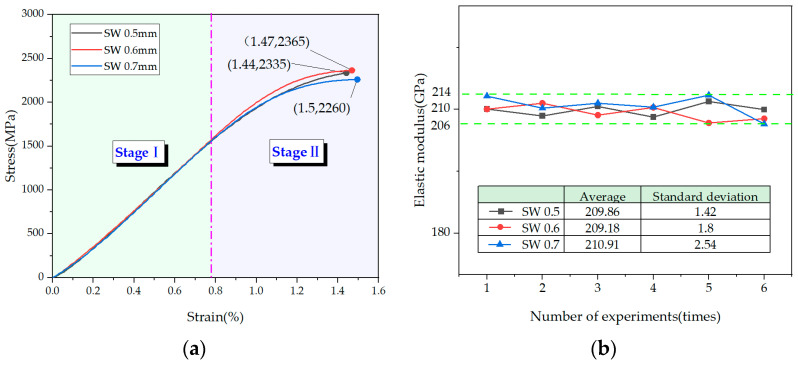
Tension of cold-drawn non-alloy steel wire for springs: (**a**) stress–strain curves of cold-drawn non-alloy steel wire for springs with different diameters.; (**b**) analysis of elastic modulus of steel wire.

Tensile tests were conducted on a specimen of cold-drawn non-alloy steel wire for springs, and the stress–strain characteristics of the steel wire during the tensile process were collected using an extensometer. The test results were as follows:

As shown in Figure 6a, the two stages of the stress–strain curve of cold-drawn non-alloy steel wire for springs are as follows: Stage I is the elastic stage, and the strain is generally 0–0.8%. Stage II is the strengthening stage, and the strain is generally 0.8–1.5%. The maximum strain of cold-drawn non-alloy steel wire for springs is between 1.4% and 1.5%, and the maximum stress is between 2200 and 2400 MPa. The maximum stress and strain of the steel wires with different diameters were roughly consistent.

Stage I is the main basis for calculating the elastic modulus of the steel wire. According to the stress–strain curve of the elastic stage of steel wires with different diameters, the least square method is adopted to fit the stress–strain curve of the elastic stage. Subsequently the elastic modulus of 0.5 mm, 0.6 mm, and 0.7 mm steel wires that fluctuate around 210 GPa can be obtained, as shown in Figure 6b. Therefore, it can be considered that the elastic modulus of the steel wire is 210 GPa.

### 3.2. Tensile Test Results of Polyethylene Geogrid Belt

**Figure 7:**
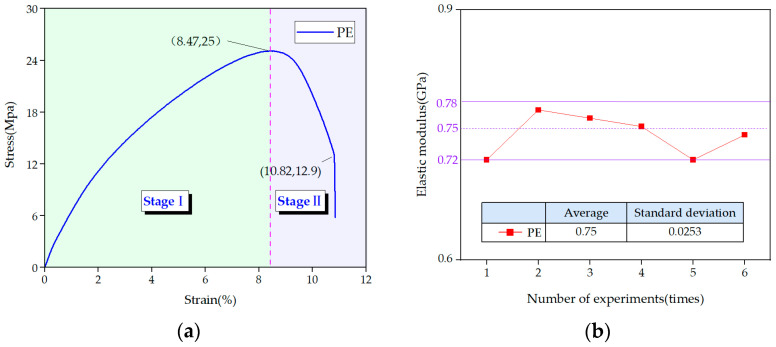
Tension of the polyethylene geogrid belt: (**a**) stress–strain curve of polyethylene geogrid belt; (**b**) distribution characteristics of the elastic modulus of the polyethylene geogrid belt.

The tensile test was conducted on a sample of the polyethylene geogrid belt in Table 1, and the stress and strain characteristics of the polyethylene geogrid belt during the tensile process were collected using an extensometer. The test results were as follows:

According to Figure 7a, the stress–strain curve of the polyethylene geogrid belt can be divided into the following two stages: Stage I is the strengthening stage, and the strain is 0–8.47%. Stage II is a local deformation stage with a strain of 8.47–10.82%. The maximum strain of the polyethylene geogrid belt reached 10.82%, and the maximum stress reached 25 MPa.

According to the standard [22,23], the tensile elastic modulus of a polyethylene material can be calculated using the chord modulus. Through the tension test of multiple sets of polyethylene geogrid belts, the elastic modulus calculated by fitting fluctuates around 0.75 GPa, as shown in Figure 7b. Therefore, the elastic modulus of the polyethylene geogrid belt was considered to be 0.75 GPa.

### 3.3. Tensile Test Results of Steel-Plastic Compound Geogrid-Reinforced Belt

The tensile test was performed on the specimens of the steel–plastic compound geogrid-reinforced belt as listed in Table 1, and the stress–strain characteristics of the steel–plastic compound geogrid-reinforced belt during the tensile process were measured using an extensometer. The calculated stress area of the steel–plastic compound geogrid-reinforced belt equals the cross-sectional area of the reinforced belt, which was obtained from the data in Table 1.

#### 3.3.1. Relationship between Stress–Strain and Tension of the Steel–Plastic Compound Geogrid-Reinforced Belt

**Figure 8:**
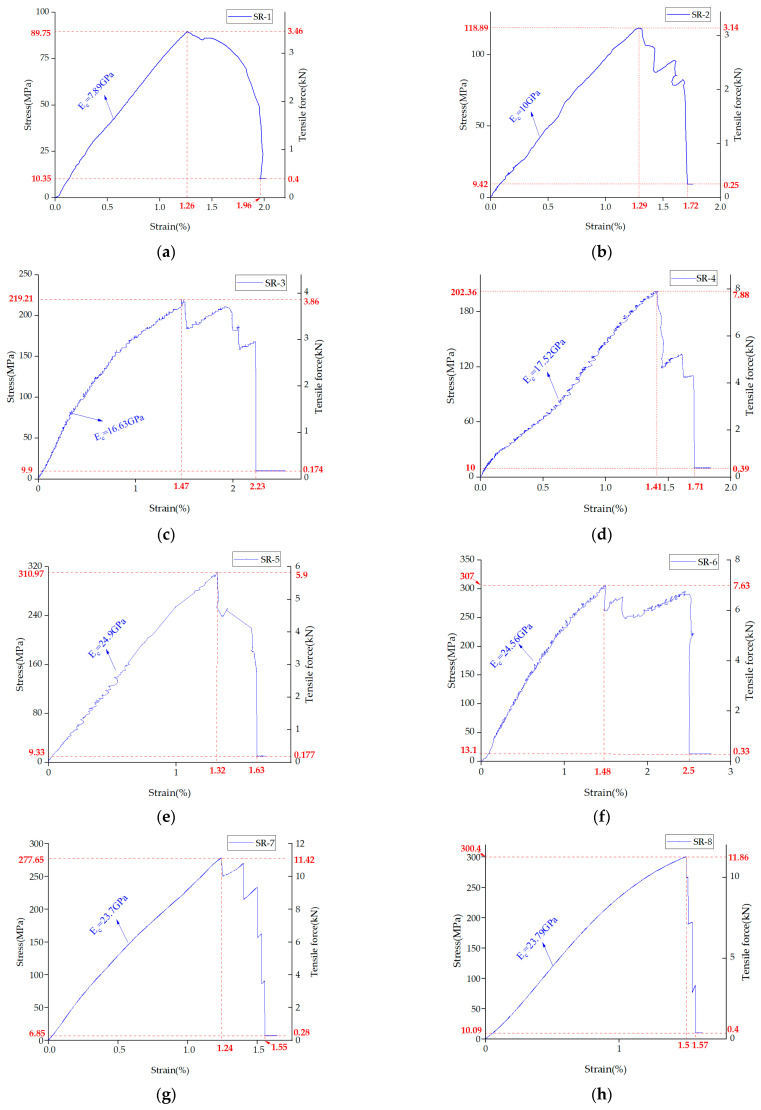

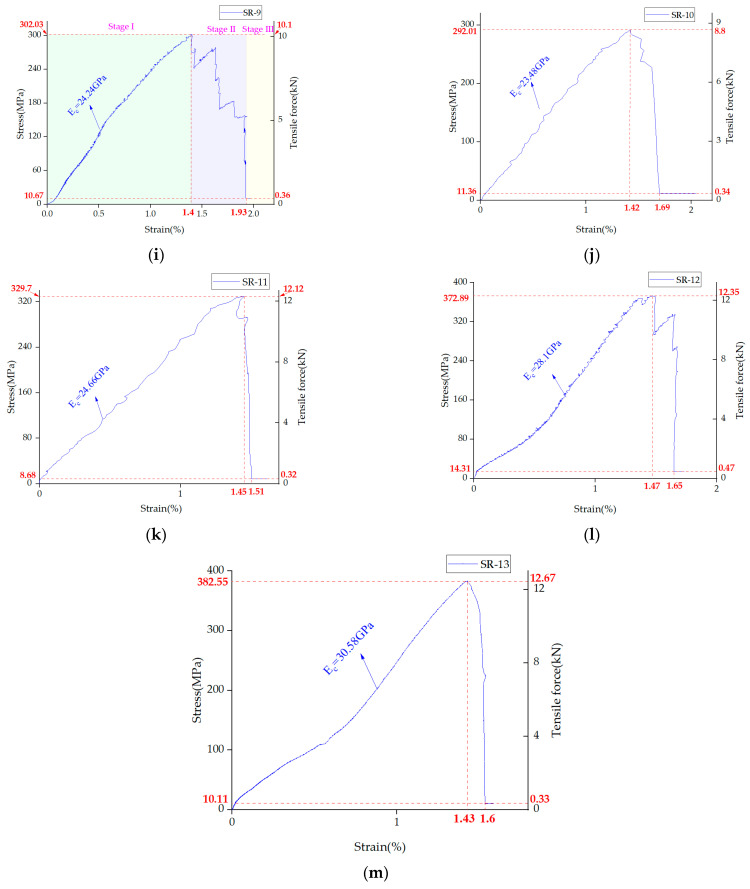
Tensile stress–strain and tensile curve of steel–plastic compound geogrid-reinforced belt: (**a**–**m**) SR-1 to SR-13.

Taking Figure 8i as an example, the tensile curves of the steel–plastic compound geogrid-reinforced belt can be divided into three stages: composite adjustment (stage I), steel wire breaking (stage II), and residual deformation (stage III). The boundary between stages I and stage II is at the position where the steel wires break for the first time (point A in Figure 8i). The boundary between stages II and III is at the position where the steel wire is completely broken in the reinforced belt (point B in Figure 8i).

The composite adjustment stage (stage I) is the dominant stress stage of the steel wire in the steel–plastic compound geogrid-reinforced belt. In this stage, the stress–strain curve of the steel–plastic compound geogrid-reinforced belt shows a fluctuating upward trend, and it fails to show the obvious elastic stage and strengthening stage in the stress-strain curve of single steel wire. This difference is due to the large number of steel wires in the steel–plastic compound geogrid-reinforced belt. When the steel wires are subjected to tension, they are not in the same state of force and there is a coordinated force between the steel wires.

The steel wire breaking stage (stage II) is a stage in which the strain of the reinforced belt exceeds the limit strain of the steel wire and the steel wire breaks. At this stage, all steel wires are pulled apart but not simultaneously, and progressive breaking occurs. Once broken, after a certain strain energy accumulation, some steel wires break again until all the steel wires are completely broken. This breaking behavior is similar to the reason for the composite adjustment stage (stage I). The steel wires in the reinforced belt are not all in the same straight state during the machining process, and some steel wires bend, resulting in incomplete cooperative deformation between the steel wires. Some steel wires are forced first, and the other steel wires are forced subsequently, resulting in a progressive fracture of the steel wires. This is also one of the reasons for the lower value of the first breaking strain of the steel wire in the reinforced belt than that of the single steel wire as well as the lower value of the tensile strength of the reinforced belt than the total tensile strength value of each steel wire.

The residual deformation stage (stage III) was completely affected by the polyethylene geogrid belt. At this stage, the stress of the steel–plastic compound geogrid-reinforced belt was between 7 and 14 MPa, and the mechanical effect of the polyethylene geogrid belt was evidently small.

From Figure 8, it can be seen that the first breaking strain of the steel wire in the steel–plastic compound geogrid-reinforced belt generally occurs between 1.2% and 1.5%, and 70% of the breaking events are concentrated in the strain of 1.4–1.5%. The final fracture strain generally occurs between 1.5% and 2.5%, and 70% of the fracture events are concentrated in the strain of 1.5–1.8%. The strain characteristics of steel–plastic compound geogrid-reinforced belts are consistent with those of a single steel wire.

#### 3.3.2. Influence of Steel Wire Specification and Root Number on the Tensile Strength of the Steel-Plastic Compound Geogrid-Reinforced Belt

1. Effect of Steel Wire Diameter in the Steel-plastic Compound Geogrid-Reinforced Belt on its Tensile Strength

**Figure 9:**
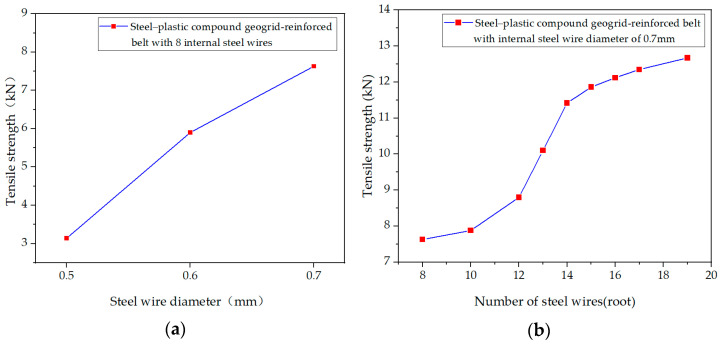
Influence of (**a**) steel wire diameter and (**b**) steel wire number on the tensile strength of the steel–plastic compound geogrid-reinforced belt.

To study the influence of the steel wire diameter on the tensile strength of steel–plastic compound geogrid-reinforced belt, SR-2, SR-5, and SR-6 groups were selected. The diameters of steel wires in the three groups were 0.5 mm, 0.6 mm, and 0.7 mm, respectively, and the number of steel wires was 8. The influence of the steel wire diameter on the tensile strength of the reinforcement was plotted according to the tensile test results of the three groups, as shown in Figure 9a. It can be seen that the tensile strength of the steel–plastic compound geogrid-reinforced belt is proportional to the diameter of the steel wire in the reinforced belt.

2. Effect of the Number of Steel Wire Roots in the Steel–Plastic Compound Geogrid-Reinforced Belt on its Tensile Strength

To study the effect of steel wire number on the tensile strength of steel–plastic compound geogrid-reinforced belt, nine experimental groups (SR-6, SR-4, SR-10, SR-9, SR-7, SR-8, SR-11, SR-12, SR-13) were selected. The numbers of steel wires in the nine groups were 8, 10, 12, 13, 14, 15, 16, 17, and 19, respectively, and the diameters of the steel wires were 0.7 mm. The influence of the steel wire number on the tensile strength of the reinforced belt is drawn as a curve according to the tensile test results of the three groups of steel–plastic compound geogrid-reinforced belts, as shown in Figure 9b. It can be seen that the tensile strength of the steel–plastic compound geogrid-reinforced belt is proportional to the number of steel wires in the reinforced belt.

#### 3.3.3. Elastic Modulus Analysis of Steel–Plastic Grille

We consider the tensile deformation characteristics of a single steel wire and the coordinated force between them when the steel wire in the reinforced belt is pulled. The smoother position in the middle of the stress–strain curve is selected before the reinforced belt reaches the maximum stress as the basis for selecting the elastic modulus *E_c_* of the steel–plastic compound geogrid-reinforced belt. Finally, the least squares method is used to perform regression fitting, and the tensile elastic modulus *E_c_* value of the steel–plastic compound geogrid-reinforced belt with different reinforcement ratios is obtained, as shown in Table 5.

## 4. Minimum and Optimal Reinforcement Ratios of Steel Wire in Steel–Plastic Compound Geogrid-Reinforced Belt

### 4.1. Minimum Reinforcement Ratio of Steel Wire for Steel-Plastic Compound Geogrid-Reinforced Belt

**Figure 10:**
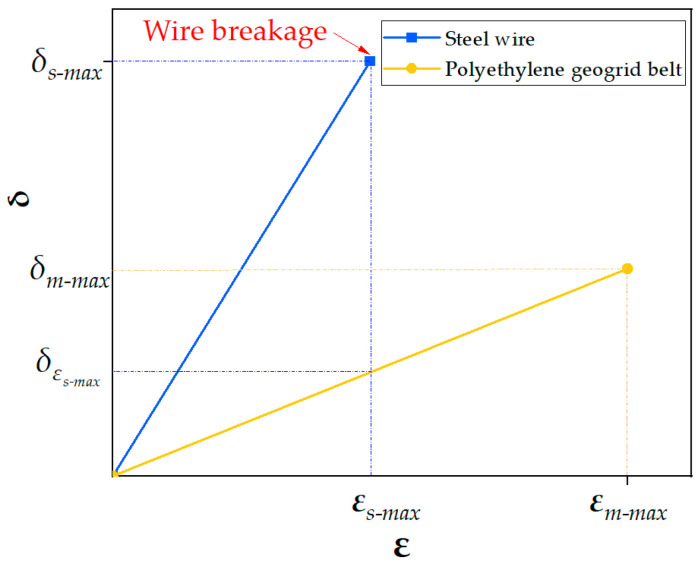
Stress–strain relationship between steel wire and polyethylene geogrid belt.

According to the tensile test results of the steel–plastic compound geogrid-reinforced belt, because the elongation of the steel wire is less than that of the polyethylene material, the steel wire breaks first during the test. Because the steel wire in the reinforced belt has the same specifications, it is assumed that each steel wire has the same strength. When the tensile force of the reinforced belt increases continuously, the deformation of the reinforced belt also accumulates continuously. Until the ultimate strain *ε_s*-max*_* of the steel wire is reached, the tensile stress of the steel wire reaches its maximum *δ_s*-max*_*, and then the steel wire is broken. Before the breaking of the steel wire, the steel wire and the polyethylene outer coating still exhibit synergistic deformation (both the strains are *ε_s*-max*_*), and the polyethylene outer coating also bears a certain stress (*δ_s*-max*_*), as shown in Figure 10.

Li et al. [24,25] studied the FRP composite theory using the stress–strain analysis method of a fiber and matrix, which provided an important reference for the composite strength analysis of steel–plastic compound geogrid-reinforced belt. When the steel–plastic compound geogrid-reinforced belt is subjected to longitudinal tensile force, the tensile force is regularly distributed in the cross section of the steel–plastic compound geogrid-reinforced belt according to the stress–strain relationship between the steel wire and polyethylene material in the reinforced belt.

Under the action of tension *P*, the steel–plastic compound geogrid-reinforced belt will have the following rules:

(2) $$P=S_{c}\times \delta_{c}=S_{s}\times \delta_{s}+S_{m}\times \delta_{m}$$

where *S_c_* and *δ_c_* are the cross-sectional area and the ideal stress of the steel–plastic compound geogrid-reinforced belt, respectively; *S_s_* and *δ_s_* are the total area and the stress of the steel wire, respectively; and *S_m_* and *δ_m_* are the total areas and the stress of the pure polyethylene geogrid belt, respectively.

The following equation can be obtained from Equation (2).

(3) $$\delta_{c}=\frac{S_{s}}{S_{c}}\times \delta_{s}+\frac{S_{m}}{S_{c}}\times \delta_{m}$$

The known conditions are as follows:

(4) $$1=\lambda_{s}+\lambda_{m}=\frac{S_{s}}{S_{c}}+\frac{S_{m}}{S_{c}}$$

where *λ_s_* is the steel wire reinforcement ratio, and *λ_m_* is the ratio of the cross-sectional area of the polyethylene coating to that of the steel–plastic compound geogrid-reinforced belt.

Substituting Equation (4) into Equation (3), we obtain the following equation.

(5) $$\delta_{c}=\lambda_{s}\times \delta_{s}+\left(1-\lambda_{s}\right)\times \delta_{m}$$

When the strain of the reinforced belt reaches the maximum strain of the steel wire, that is, *ε_s_* = *ε_s_*_-max_, and *δ_s_* = *δ_s_*_-max_, $\delta_{m}=\delta_{\varepsilon }{}_{{}_{s-\max}}$. Equation (5) can be modified to

(6) $$\delta_{c}=\lambda_{s}\times \delta_{s-\max}+\left(1-\lambda_{s}\right)\times \delta_{\varepsilon }{}_{{}_{s-\max}}$$

where *δ_s_*_-max_ is the maximum stress of the steel wire, and $\delta_{\varepsilon }{}_{{}_{s-\max}}$ is the stress value of polyethylene corresponding to the maximum strain of the steel wire.

Shen et al. [26] proposed that the strength of composite materials should be greater than that of pure matrix materials, considering the main role of fibers in composites. When the strain of the steel–plastic compound geogrid-reinforced belt reaches the maximum strain of the steel wire and the steel wire plays a reinforcing role, the strength of the steel–plastic compound geogrid-reinforced belt *δ_c_* should be greater than that of the pure polyethylene geogrid belt *δ_m_*_-max_, that is,

(7) $$\delta_{c}>\delta_{m-\max}$$

where *δ_m_*_-max_ is the maximum strength of the pure polyethylene geogrid belt.

The following equation can be obtained from Equations (6) and (7).

(8) $$\lambda_{s}>\frac{\delta_{m-\max}-\delta_{\varepsilon }{}_{{}_{s-\max}}}{\delta_{s-\max}-\delta_{\varepsilon }{}_{{}_{s-\max}}}$$

Assuming that the minimum reinforcement ratio is $\lambda_{s}{}_{-cr}$ when the steel wire plays a leading role, then $\lambda_{s}{}_{-cr}=\frac{\delta_{m-\max}-\delta_{\varepsilon }{}_{{}_{s-\max}}}{\delta_{s-\max}-\delta_{\varepsilon }{}_{{}_{s-\max}}}$. Then, when $\lambda_{s}>\lambda_{s}{}_{-cr}$, the steel wire strengthens the composite effect of the reinforced belt. When $\lambda_{s}=\lambda_{s}{}_{-cr}$, the steel wire does not strengthen or weaken the composite effect of the reinforced belt. When $\lambda_{s}<\lambda_{s}{}_{-cr}$, the steel wire does not strengthen the composite effect of the steel–plastic belt but weakens the pure polyethylene geogrid belt.

The parameter values listed in Table 6 were obtained by tensile testing. The minimum reinforcement ratio $\lambda_{s}{}_{-cr}$ is slightly different (0.2‰) owing to the different steel wire diameters. Compared with the steel wire diameters of 0.5 mm, 0.6 mm, and 0.7 mm, the influence of steel wire diameter on the minimum reinforcement ratio can be ignored. Therefore, it can be considered that the minimum reinforcement ratio is 0.63%; that is, when the steel wire plays a leading role, its minimum reinforcement ratio in the steel–plastic compound geogrid should exceed 0.63%.

### 4.2. Optimal Reinforcement Ratio of Steel Wire for Steel–Plastic Compound Geogrid-Reinforced Belt

**Figure 11:**
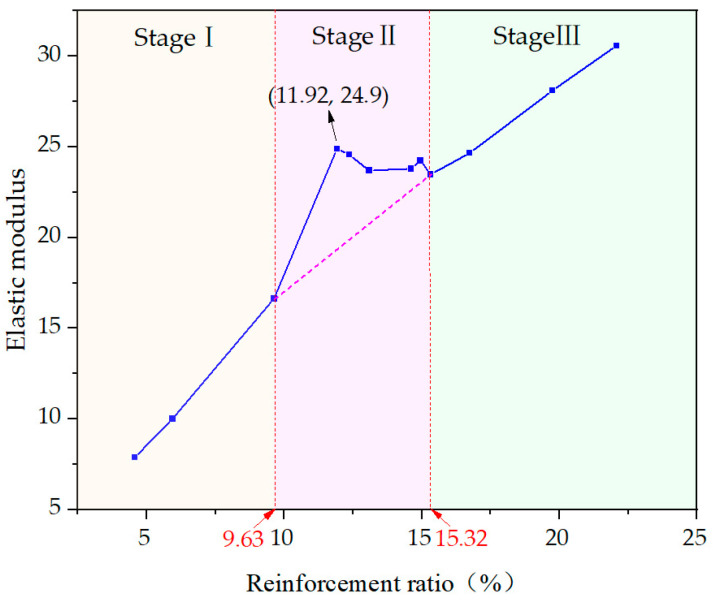
Elastic modulus and reinforcement ratio curve.

According to the calculation results of the elastic modulus of the steel–plastic compound geogrid-reinforced belt, the relationship between the elastic modulus and reinforcement ratio is obtained as shown in Figure 11.

The elastic modulus and reinforcement ratio are generally positively correlated and nonlinear. According to the changing trend of the curve, I, II, and III can be divided into three stages.

Stage I is the initial stage of stable growth, and the relationship between the elastic modulus and reinforcement ratio tends to develop linearly. The elastic modulus increases with increasing reinforcement ratio.

Stage II is the stage with an evident mid-term gain, and the curve change rate increases rapidly. When the reinforcement ratio reaches 11.92%, the elastic modulus reaches the maximum, and then the fluctuation decreases slowly.

Stage III is the later stage of steady growth and maintains the same curve change rate as stage I.

From the above analysis, it can be seen that when the reinforcement ratio is 11.92%, the steel wire consumption of the steel–plastic compound geogrid-reinforced belt is the most economical and efficient. Therefore, 11.92% is determined as the optimal reinforcement ratio.

## 5. Conclusions

The tensile mechanical properties of a steel wire, polyethylene geogrid belt, and steel–plastic compound geogrid-reinforced belt and the composite characteristics of steel–plastic compound geogrid-reinforced belt were analyzed and studied through tensile testing. Based on the experimental data, the minimum reinforcement ratio and optimal reinforcement ratio of steel wire in the steel–plastic compound geogrid-reinforced belt were analyzed, and the following conclusions were drawn.

1. The tensile stress–strain curve of the steel wire was divided into an elastic deformation stage and a strengthening stage. According to the tensile test data of the steel wire, the elastic modulus, maximum stress, and maximum strain of steel wires with different diameters were roughly the same. The elastic modulus of the steel wire was 210 GPa; the maximum stress and maximum strain were between 2200 and 2400 MPa, and between 1.4% and 1.5%, respectively. The tensile stress–strain curve of the polyethylene geogrid belt can be divided into the strengthening and local deformation stages. According to the tensile test data of the polyethylene geogrid belt, the elastic modulus of the polyethylene geogrid belt was 0.75 GPa; maximum stress was 25 MPa, and the maximum strain was 10.82%.
2. The tensile stress–strain curve of the steel–plastic compound geogrid-reinforced belt is divided into a composite adjustment stage, steel wire breaking stage, and residual deformation stage. In the composite adjustment stage, the steel wire plays a dominant role in the force of the belt. In the fracture stage of the steel wire, the fracture form of the steel wire is progressive. The first fracture strain of steel wire in this belt generally occurs between 1.2% and 1.5%, and the final fracture strain generally occurs between 1.5% and 2.5%. In the residual deformation stage, the mechanical properties of steel–plastic compound geogrid-reinforced belts are completely attributed to the polyethylene materials. The tensile strength of this belt is proportional to the diameter and number of steel wires in the reinforced belt.
3. The minimum reinforcement ratio of steel wire in a steel–plastic compound geogrid-reinforced belt is 0.63%, as determined by mathematical derivation and calculation. According to the tensile test results, the optimal reinforcement ratio of steel wire in this belt is 11.92%. The results of this study pave the way for optimizing the material properties of steel–plastic compound geogrid as well as its engineering reinforcement performance and provide a reliable scientific basis for further research.

## 6. Discussion

In order to study the tensile mechanical properties of steel–plastic compound geogrid-reinforced belt, tensile mechanical tests of steel wire, polyethylene geogrid belt, and steel–plastic compound geogrid-reinforced belt were carried out, and the minimum and optimal reinforcement ratios of steel–plastic compound geogrid-reinforced belts were determined. However, the durability of steel–plastic compound geogrids is still unclear. Therefore, the durability of steel–plastic compound geogrid and the mechanical loss of steel–plastic compound geogrid under different engineering application environments can be systematically studied in the future.

Several models have been used in previous studies to explain the composite mechanical behavior of steel–plastic compound geogrid-reinforced belts. However, owing to the incompatibility of steel wire in the reinforced belt and the influence of single steel wire breakage on the overall reinforced belt, the prediction accuracy of these models for the trend of the overall mechanical properties of the reinforced belt is low. Therefore, it is necessary to establish a constitutive model of a steel–plastic compound geogrid-reinforced belt to measure the change in the tensile mechanical behavior of the steel–plastic compound geogrid.

Considering these two points, we will conduct further research based on the results of the present study.

## Figures and Tables

**Table 1:** Design specifications of experimental group.

Material Type	Experimental Group Number	Wire Diameter (mm) *d*	Number of Steel Wires (Root) *n*	Sectional Area Size of the Reinforced Belt		Reinforcement Ratio *λ_S_* (%)
				Width (mm) *b*	Thickness (mm) *h*	
Cold-drawn non-alloy steel wire for springs	SW0.5	0.5	/	/	/	/
	SW0.6	0.6	/	/	/	/
	SW0.7	0.7	/	/	/	/
Polyethylene geogrid belt	PE	/	/	8.1	1.6	/
Steel–plastic compound geogrid reinforced belt	SR-1	0.5	9	15.8	2.44	0.0458
	SR-2	0.5	8	13.9	1.9	0.0594
	SR-3	0.6	6	10.12	1.74	0.0963
	SR-4	0.7	10	17.7	2.2	0.0988
	SR-5	0.6	8	10.78	1.76	0.1192
	SR-6	0.7	8	10.92	2.28	0.1236
	SR-7	0.7	14	19.22	2.14	0.1309
	SR-8	0.7	15	19.94	1.98	0.1461
	SR-9	0.7	13	15.2	2.2	0.1495
	SR-10	0.7	12	15.22	1.98	0.1532
	SR-11	0.7	16	17.34	2.12	0.1674
	SR-12	0.7	17	18.4	1.8	0.1974
	SR-13	0.7	19	18.4	1.8	0.2207

**Table 2:** Test pre-tension values of cold-drawn non-alloy steel wires for springs.

Experimental Group Number	Fracture Stress of Steel Wire (MPa) *σ_s_*	Maximum Force of Steel Wire (N) *F_s_* = *σ_s_* × *S*_1_	$\mathrm{Pre}-\mathrm{Tension}\;\mathrm{Values}\;(N)\;F_{l_{s}}\;=\;F_{s}\times 1\%$
SW0.5	2000–2400	390–470	3.90–4.70
SW0.6		565–678	5.65–6.78
SW0.7		769–923	7.69–9.23

**Table 3:** Test pre-tension values of polyethylene geogrid belt.

Experimental Group Number	Maximum Force of Polyethylene Geogrid Belt (N) *F_m_*	$\mathrm{Pre}-\mathrm{Tension}\;\mathrm{Value}\;(N)\;F_{l_{m}}\;=\;F_{m}\times 1\%$
PE	280–350	2.8–3.5

**Table 4:** Test pre-tension values of steel–plastic compound geogrid-reinforced belt.

Experimental Group Number	Maximum Force of Single Steel Wire (N) *F_s_*	Maximum Force Estimation of Reinforced Belt (N) *F_c_* = *F_s_* × *n*	$\mathrm{Pre}-\mathrm{Tension}\;\mathrm{Value}\;\mathrm{of}\;\mathrm{Reinforced}\;\mathrm{Belt}\;(N)\;F_{l_{c}}\;=\;F_{c}\times 1\%$	Experimental Group Number	Maximum Force of Single Steel Wire (N) *F_s_*	Maximum Force Estimation of Reinforced Belt (N) *F_c_* = *F_s_* × *n*	$\mathrm{Pre}-\mathrm{Tension}\;\mathrm{Value}\;\mathrm{of}\;\mathrm{Reinforced}\;\mathrm{Belt}\;(N)\;F_{l_{c}}\;=\;F_{c}\times 1\%$
SR-1	390–470	3510–4230	35.10–42.30	SR-8	769–923	11,535–13,845	115.35–138.45
SR-2	390–470	3120–3760	31.20–37.60	SR-9	769–923	9997–11,999	99.97–119.99
SR-3	565–678	3390–4068	33.90–40.68	SR-10	769–923	9228–11,076	92.28–110.76
SR-4	769–923	7690–9230	76.90–92.30	SR-11	769–923	12,304–14,768	123.04–147.68
SR-5	565–678	4520–5424	45.20–54.24	SR-12	769–923	13,073–15,691	130.73–156.91
SR-6	769–923	6152–7384	61.52–73.84	SR-13	769–923	14,611–17,537	146.11–175.37
SR-7	769–923	10,766–12,922	107.66–129.22	/	/	/	/

**Table 5:** Tensile modulus *E_c_* value of steel–plastic compound geogrid-reinforced belt with different reinforcement ratios.

Test Group	Modulus of Elasticity (GPa)	Test Group	Modulus of Elasticity (GPa)	Test Group	Modulus of Elasticity (GPa)
SR-1	7.89	SR-6	24.56	SR-11	24.66
SR-2	10	SR-7	23.7	SR-12	28.1
SR-3	16.63	SR-8	23.79	SR-13	30.58
SR-4	17.52	SR-9	24.24	/	/
SR-5	24.9	SR-10	23.48	/	/

**Table 6:** Minimum reinforcement ratio when steel wire plays a leading role.

Material Type		$\delta_{m-\max}$ (MPa)	$\varepsilon_{s-\max}$ (%)	$\delta_{s-\max}$ (MPa)	$\delta_{\varepsilon }{}_{{}_{s-\max}}$ (MPa)	$\lambda_{s-cr}$ (%)
Polyethylene geogrid belt		25	/	/	/	/
Steel wire	0.5 mm	/	1.44	2335	10.30	0.63
	0.6 mm	/	1.47	2365	10.60	0.61
	0.7 mm	/	1.5	2260	10.91	0.63

## Data Availability

The data presented in this study are available upon request from the corresponding author.
